# Quality of care for postpartum hemorrhage: A direct observation study in referral hospitals in Kenya

**DOI:** 10.1371/journal.pgph.0001670

**Published:** 2023-03-02

**Authors:** Emma Clarke-Deelder, Kennedy Opondo, Emmaculate Achieng, Lorraine Garg, Dan Han, Junita Henry, Moytrayee Guha, Alicia Lightbourne, Jennifer Makin, Nora Miller, Brenda Otieno, Anderson Borovac-Pinheiro, Daniela Suarez-Rebling, Nicolas A. Menzies, Thomas Burke, Monica Oguttu, Margaret McConnell, Jessica Cohen

**Affiliations:** 1 Department of Global Health and Population, Harvard T. H. Chan School of Public Health, Boston, MA, United States of America; 2 Department of Epidemiology and Public Health, Swiss Tropical and Public Health Institute, Allschwil, Switzerland; 3 Kisumu Medical and Education Trust, Kisumu, Kenya; 4 Vayu Global Health Foundation, Boston, MA, United States of America; 5 Department of Emergency Medicine, Global Health Innovation Laboratory, Massachusetts General Hospital, Boston, MA, United States of America; 6 Lee Kuan Yew School of Public Policy, National University of Singapore, Singapore, Singapore; 7 Economics Department, Massachusetts Institute of Technology, Cambridge, MA, United States of America; 8 Brown University, Providence, RI, United States of America; 9 Duke University, Durham, North Carolina, United States of America; 10 Department of Obstetrics and Gynecology, University of Pittsburgh Medical Center, Pittsburgh, PA, United States of America; 11 Department of Obstetrics and Gynecology, School of Medical Sciences, University of Campinas, Campinas (SP), Brazil; 12 Harvard Medical School, Boston, MA, United States of America; University of Washington, UNITED STATES

## Abstract

Postpartum hemorrhage (PPH) is the leading cause of maternal mortality in Kenya. The aim of this study was to measure quality and timeliness of care for PPH in a sample of deliveries in referral hospitals in Kenya. We conducted direct observations of 907 vaginal deliveries in three Kenyan hospitals from October 2018 through February 2019, observing the care women received from admission for labor and delivery through hospital discharge. We identified cases of “suspected PPH”, defined as cases in which providers indicated suspicion of and/or took an action to manage abnormal bleeding. We measured adherence to World Health Organization and Kenyan guidelines for PPH risk assessment, prevention, identification, and management and the timeliness of care in each domain. The rate of suspected PPH among the observed vaginal deliveries was 9% (95% Confidence Interval: 7% - 11%). Health care providers followed all guidelines for PPH risk assessment in 7% (5% - 10%) of observed deliveries and all guidelines for PPH prevention in 4% (3% - 6%) of observed deliveries. Lowest adherence was observed for taking vital signs and for timely administration of a prophylactic uterotonic. Providers did not follow guidelines for postpartum monitoring in any of the observed deliveries. When suspected PPH occurred, providers performed all recommended actions in 23% (6% - 40%) of cases. Many of the critical actions for suspected PPH were performed in a timely manner, but, in some cases, substantial delays were observed. In conclusion, we found significant gaps in the quality of risk assessment, prevention, identification, and management of PPH after vaginal deliveries in referral hospitals in Kenya. Efforts to reduce maternal morbidity and mortality from PPH should emphasize improvements in the quality of care, with a particular focus on postpartum monitoring and timely emergency response.

## Introduction

Postpartum hemorrhage (PPH) is the leading cause of maternal mortality worldwide [1]. The highest burden is in low- and middle-income countries (LMICs) [2].

Effective interventions exist to prevent and treat PPH during antenatal, delivery and postpartum care [3]. However, even with growing rates of delivery in health facilities [4] and widespread availability of medicines such as uterotonic drugs [5], the burden of PPH remains high in LMICs. This suggests that more attention is needed to the quality and timeliness of care that patients receive during their delivery hospitalization.

To inform efforts to improve quality of care, it is important to understand gaps in the quality of PPH prevention, identification, and management. A growing literature has documented significant gaps in the quality of maternity care in LMICs [6–9]. However, since past studies have mainly focused on routine care during labor and delivery, there is a need for more evidence on gaps in the quality of care specifically related to PPH including postpartum monitoring and emergency management. Moreover, given that delays in responding to PPH can contribute to morbidity and mortality, there is also a need for evidence on timeliness of care. The few studies that have assessed quality of care specifically for PPH in LMICs have found important gaps such as limited monitoring of vaginal bleeding and uterine tone [10], and under-use of [11] and delayed administration of [12] therapeutic uterotonics.

Research into the quality of care for PPH is particularly important in settings with high maternal mortality such as Kenya. Kenya has a maternal mortality ratio of 338 deaths per 100,000 live births, with approximately 40% of maternal deaths attributable to PPH [1]. In recent years, Kenya has implemented several reforms to increase access to maternity care, including a voucher program for subsidized maternity care, the introduction of free maternity care in public health facilities in 2013, and the expansion of free maternity care to private facilities through the Linda Mama program in 2016. These policies have led to significant increases in the rate of facility-based delivery [13,14], despite several challenges in implementation that affected access for some potential beneficiaries [15]. There have also been significant efforts to scale up effective interventions for PPH prevention and treatment in health facilities in Kenya including training health care providers on Active Management of the Third Stage of Labor and increasing the coverage of effective uterotonic medicatoins [16,17]. However, mortality from PPH remains high despite these efforts and a recent review of maternal mortality in Kenya identified quality of care as a key challenge [18]. As a result, there is an increased focus on understanding gaps in quality and identifying interventions to address them. With the goal of improving maternal health care quality, the Ministry of Health recently launched a series of new guidelines and standards for obstetric and perinatal care [19].

The aim of this study was to measure the quality and timeliness of PPH care in referral hospitals in Kenya in order to inform efforts to improve quality of care.

## Materials and methods

### Ethics statement

The protocol for this study was approved by the Harvard University Institutional Review Board (IRB18-1342), the Ethics and Research Committee of the Jaramogi Oginga Odinga Teaching and Referral Hospital in Kisumu, Kenya (ERC.IB/VOL.1/507), and the National Commission for Science, Technology and Innovation in Kenya (NACOSTI/P/19/68231/30907). The study was also approved by the study hospitals and the County Health Management Teams in the locations where data were collected.

Written consent was obtained from all participating patients and verbal consent was obtained from all participating health care providers. The IRB waived the requirement for parent or guardian consent for participants aged 16–17 as a result of the national guidelines in Kenya [20]. To limit the identifying information that we recorded and kept on health care providers, verbal consent was sought (and approved); this helped to ensure that providers’ identifying information could not be linked to their actions in patient care if they were part of a government investigation. A list of enrolled health care providers was maintained during data collection but destroyed at the end of the data collection period.

### Study design

We conducted a cross-sectional study using direct observations of labor, delivery, and postpartum care.

### Study setting and population

This study was implemented in three hospitals in Nairobi and western Kenya. The study hospitals each had between approximately 500 and 1500 deliveries per month in 2018. They are all Level 5 referral hospitals in the Kenyan system, meaning that they serve as regional referral and training hospitals. All three have capacity for comprehensive emergency obstetric care [21], including blood transfusion and Caesarean section. Caesarean sections make up approximately 25% of the deliveries that occur in the study hospitals. In these hospitals, labor, delivery, and postpartum care is primarily provided by nurse-midwives and students. More senior clinicians, including Medical Officers, and consultant physicians such as obstetrician-gynecologists and pediatricians, make periodic rounds and are available on-call to assist in emergencies.

### Study sample

Our study included pregnant women aged 16 or over who delivered vaginally and provided informed consent. Women meeting eligibility criteria were approached for consent upon arrival to the hospital. Referred patients who had not yet delivered by the time they arrived at the study hospital were included, as were patients who required surgical intervention for PPH management. Women who delivered by caesarean section were excluded.

The sampling strategy was to enroll all eligible women during the data collection period in each hospital.

### Data collection

We developed an observation checklist to measure timely adherence to WHO and Kenyan clinical guidelines for PPH care [22–25] based on checklists used in previous studies [26–28].

We then recruited a team of Kenyan clinicians to conduct clinical observations using the checklist. Recruitment was conducted using job boards in educational and clinical settings in the counties where the study hospitals are located. Selection was based clinical experience, data collection experience, and availability during the study period. Team members who had worked in one of the study hospitals in the past 1 year were not eligible to conduct observations in that hospital. Data collection training took place over a 2-week period in September 2018 and included classroom training on the study protocol, research ethics, and data collection tools, and practical training in a referral hospital that was not part of the study. During the practical training, multiple team members observed the same case, and observation results were discussed to ensure consistency in the data collection approach across observers. Following training, the data collection tools were revised to improve clarity and usability. 30 candidates participated in the training, and 24 were eventually hired for the study. 2 team members dropped out during the data collection process, and were replaced from the group that had been initially trained. A refresher training was conducted for the full data collection team in January 2019, partway through the data collection period.

Data collection took place from October 2018 through February 2019. Observations were conducted for three to four weeks in each of the study hospitals, based on available budget. Observations took place for 24 hours per day, with gaps for two rotating days per week. Patients were followed from admissions through discharge from the hospital or 24 hours after delivery, if PPH did not occur within 24 hours. In situations where there were too few observers to observe all patients receiving care, observers prioritized any woman with suspected PPH. If no woman had suspected PPH, observers completed their current observation before switching over to a new patient. Some observations were censored because the patient’s delivery care began before the observation period started or ended after the observation period (e.g., due to the timing of direct observation shifts or because the patient was not able to consent prior to admission or delivery).

We were unable to measure the prevalence of PPH based on clinical symptoms such as blood loss and vital signs because these symptoms were not consistently measured in the study hospitals. We did not introduce these measurements because they would have affected the documentation of quality of care. Instead, we focused on “suspected PPH,” defined as any delivery where a provider indicated concern or took action to manage potentially excessive blood loss. This could include stating concern aloud, conducting a vaginal exam in response to visual blood loss or a patient’s call for help about blood loss, or providing a therapeutic dose of uterotonic. This definition is relevant for evaluating the quality of care because providers should take action to manage PPH whenever they suspect a patient’s bleeding may be excessive, even if blood loss has not crossed the 500 ml threshold used to define PPH in many clinical guidelines [29]. Different patients can have different clinical responses to the same volume of blood loss due to physical differences such as differences in anemia status and stature [30]. Since our study followed women until they were discharged (or through 24 hours post-delivery if PPH did not occur within 24 hours), we expected to capture most cases of PPH that developed while patients were in the hospital.

In addition to observational data on adherence to clinical guidelines, we also collected data on patient, provider, and hospital characteristics. Data on patient characteristics were collected from patients’ antenatal care books, which contain information about patient history and care received during pregnancy, and from observation of the initial exam, during which patients share information about their medical history with their providers. Data on provider characteristics were collected through interviews with a convenience sample of providers. Hospital characteristics were collected through interviews with hospital staff members and visual assessment (e.g., of supply availability) using tools adapted from prior studies [21,31]. In addition, throughout the observation period, data were collected on the number and cadres of health care providers and patients present in the maternity ward.

All data were collected on paper forms and then entered into a digital database using SurveyCTO. Strict standards for data storage were followed to ensure the privacy and confidentiality of the data.

### Analysis

We measured adherence to evidence-based clinical guidelines for PPH. Indicators were developed based on three sources: (1) WHO clinical guidelines and recommendations [3,23,32]; (2) Kenyan national clinical guidelines [22]; and (3) prior studies of adherence to clinical guidelines for delivery care in Kenya and other settings [28,33]. We grouped indicators into four categories: (1) PPH risk assessment, such as asking relevant patient history; (2) PPH prevention, such as the use of prophylactic uterotonics; (3) patient monitoring for signs of PPH, such as taking vital signs and assessing blood loss; and (4) PPH management, such as administering therapeutic uterotonics and other actions that should be taken in every case of suspected PPH. The indicators and their sources are listed in S1 Table.

Using direct observation, we measured the proportion of deliveries in which each clinical guideline was followed among patients for whom the guideline was relevant, and the timing of clinical actions. The complete list of outcome variables and the patients for whom they were relevant is included in S1 Table. In the absence of specific guidelines for when many actions should be taken [22,23], we focused on describing the distribution of when actions occurred relative to the time of delivery (for routine care) or relative to the time of PPH identification (for PPH management). We defined the time of delivery as the time when the last fetus (e.g., the second twin) was fully delivered and the time of PPH identification as the time when a provider first mentioned concern about or took an action to treat suspected PPH.

We described the characteristics of the study sample and then described average guideline adherence. We estimated proportions and 95% confidence intervals using a normal approximation, with intervals truncated between 0 and 1.

Our estimate of PPH prevalence relies on the assumption that the rate of suspected PPH among the observed deliveries is the same as the rate overall, including deliveries that were censored due to the timing of the data collection period or staff capacity limitations. We estimated bounds on this estimate, relaxing this assumption.

Some data were missing because the observer left an item blank or indicated that they did not know whether an action had been taken. S2 Table shows the sample size for each indicator and the percent of observations with missing data. In our main analysis, we omitted missing data based on the assumption that data were missing completely at random. Most of the missing data were driven by pauses in the data collection period when observers had days off (two per week). The days off varied each week so that the observation team did not systematically miss a particular day of the week. Therefore, we expected these missing data to be essentially random. We examined the robustness of our findings to different assumptions about missing data. Missing data analyses are described in S1 Text.

Data analysis was conducted using Stata and R.

## Results

### Characteristics of the study sample

**Fig 1:**
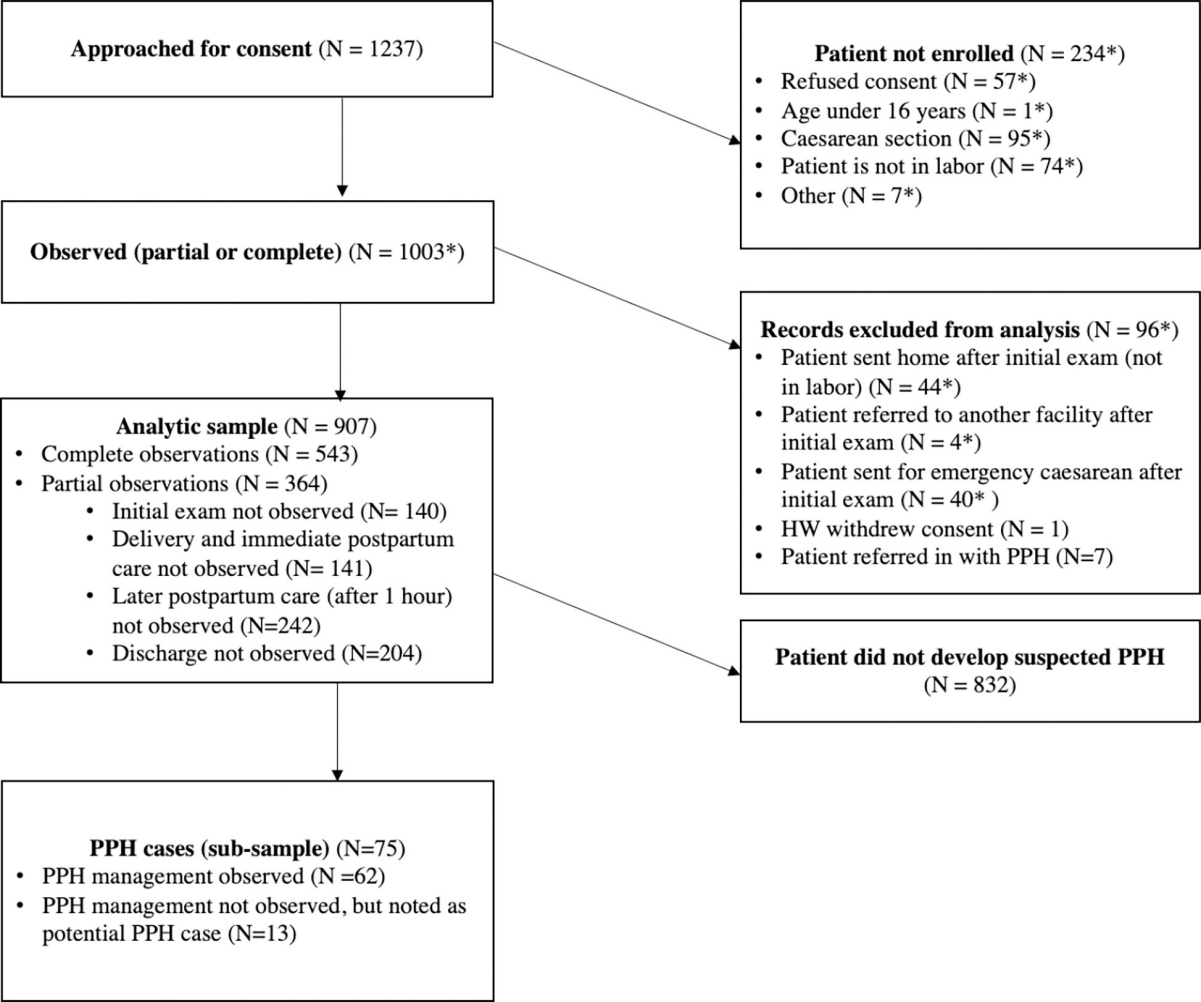
Sample flowchart. Notes: HW stands for Health Worker. PPH stands for postpartum hemorrhage. *Indicates that this number is incomplete because unobserved cases were not tracked in one of the three study hospitals.

907 deliveries met our inclusion criteria (Fig 1). Of these, 543 were fully observed (from admission through discharge from the health hospital) and the remainder were partially observed (i.e., at least one section of the delivery, such as the initial exam or later postpartum care, was not observed). The estimated prevalence of suspected PPH was 8.6% (95% CI: 6.6% - 10.6%).

Table 1 shows the characteristics of the study hospitals, the providers involved in maternity care, and the patients in the sample. The study hospitals conducted an average of 658 vaginal and 189 caesarean deliveries per month in 2018. At the time of the hospital assessment, all hospitals had oxytocin in stock; and two out of three had tranexamic acid (typically stored in the hospital pharmacy), misoprostol, and blood products in stock. The hospitals had an average of 5 blood pressure cuffs in the labor & delivery ward (though one hospital had only one cuff in the labor & delivery ward) and 2 in the postpartum ward. During the study period, a typical shift was staffed mainly by nurse-midwives (5.6 on average across the hospitals) and nursing students (average of 7.3) with an average of 1 intern and 0–1 Medical Officers or Clinical Officers present. There were no consultant physicians present at any time of day on 70% of the days during the observation period. Women in the sample attended on average four antenatal care visits during their pregnancy. In the sample, 30% of women had Haemoglobin (Hb) levels below 11.0 during their pregnancy, and 7% of women were treated for anemia during their pregnancy. Among those tested during antenatal care, 7% of women tested positive for HIV. Among those asked, 1% of women reported experiencing antepartum hemorrhage during their current pregnancy, 1% of women with past pregnancies had a history of PPH, and 3% of women with past pregnancies had had a Caesarean section.

**Table 1:** Hospital and patient characteristics.

Hospital characteristics ^3^	Mean		
*Hospital size and patient volume*			
Vaginal deliveries per month (2018)^2^	658		
Caesarean deliveries per month (2018) ^3^	188		
Number of beds in labor and delivery ward	8		
Number of beds in postpartum ward	36		
*Hospital infrastructure*, *supplies*, *and medication*^*2*^			
Has functional blood bank	100%		
Has operating theatre	100		
Number of complete delivery kits^4^	17		
Number of blood pressure cuffs in labor and delivery ward	5		
Number of blood pressure cuffs in postpartum ward	2		
Had oxytocin in stock during hospital assessment	100%		
Had misoprostol in stock during hospital assessment	67%		
Had tranexamic acid in stock during hospital assessment	67%		
Had blood products in stock during hospital assessment	67%		
*Hospital staffing*			
Number of consultants, Medical Officers or Clinical Officers during an average shift	0.5		
Number of interns during an average shift	1.0		
Number of nurse-midwives during an average shift	5.6		
Number of students during an average shift	7.3		
Percent of days when consultant is present	30%		
*Average experience level of providers (years working in maternity care)* ^*3*^			
Nurse-midwives	4.6		
Medical Officers and Clinical Officers	3.6		
Students	0.9		
**Patient demographics and PPH risk factors** ^5^	**n (%)**	**mean (SD)**	**N**
Age	-	25.3 (5.2)	859
First pregnancy	305 (35.6%)	-	856
Parity	-	1.1 (1.2)	854
HIV positive	59 (7.0%)	-	845
Number of antenatal care visits during this pregnancy	-	4.1 (1.5)	854
Received iron folic acid tablets during antenatal care	672 (78.0%)	-	861
Hemoglobin (Hb) tested during this pregnancy	767 (89.6%)	-	856
If tested, Hb result under 11.0	230 (30.0%)	-	767
Treated for anemia during this pregnancy	55 (6.4%)	-	854
APH during the current pregnancy	9 (1.1%)	-	855
History of APH during past pregnancies	14 (4.4%)	-	319
History of PPH following past deliveries	2 (0.6%)	-	319
History of caesarean section	9 (2.7%)	-	336

Notes

^1^Hospital-level statistics in this table are averaged by hospital and then summarized across hospitals. For example, the number of nurse-midwives during an average shift was first calculated for each hospital, and the experience of midwives is first averaged by hospital and then across hospitals. ^2^Source: Kenya’s Health Management Information System. ^3^Source: Hospital assessment, conducted once at the beginning of the observation period in each hospital. ^4^Includes kidney dish, cord scissors, sponge, holding forceps, episiotomy scissors, artery forceps, straight scissors, needle holder, gully pot. ^5^Patient information in this table was extracted from patients’ antenatal care booklets, with the exception of the final three rows which are based on information provided by patients during their initial examination.

### Characteristics of suspected PPH cases

Characteristics of the observed PPH cases are presented in Table 2. Based on the diagnoses stated by providers during care, the most common cause was atonic uterus (50%), followed by retained products of conception (44%), vaginal lacerations (23%), perineal lacerations (18%) and cervical lacerations (5%). PPH was typically managed by a team of providers. The most senior provider involved in PPH care was a midwife in 58% of cases. Consultant physicians were involved in PPH care in 6% of the observed cases.

**Table 2:** Characteristics of observed PPH cases.

*PPH case characteristics*	n (%)	/N
Cause (as diagnosed by provider)		62
Atonic uterus	31 (50%)	62
Retained products of conception	27 (44%)	62
Vaginal laceration	14 (23%)	62
Perineal laceration	11 (18%)	62
Cervical laceration	3 (5%)	62
Highest cadre provider involved in response^1^		
Student	5 (10%)	50
Nurse-midwife	29 (58%)	50
Clinical Officer or Medical Officer	13 (26%)	50
Consultant	3 (6%)	50

^1^Defined as the highest cadre provider who provided any care for the patient from the time when PPH was identified until bleeding was resolved.

### Adherence to guidelines for PPH care

**Fig 2:**
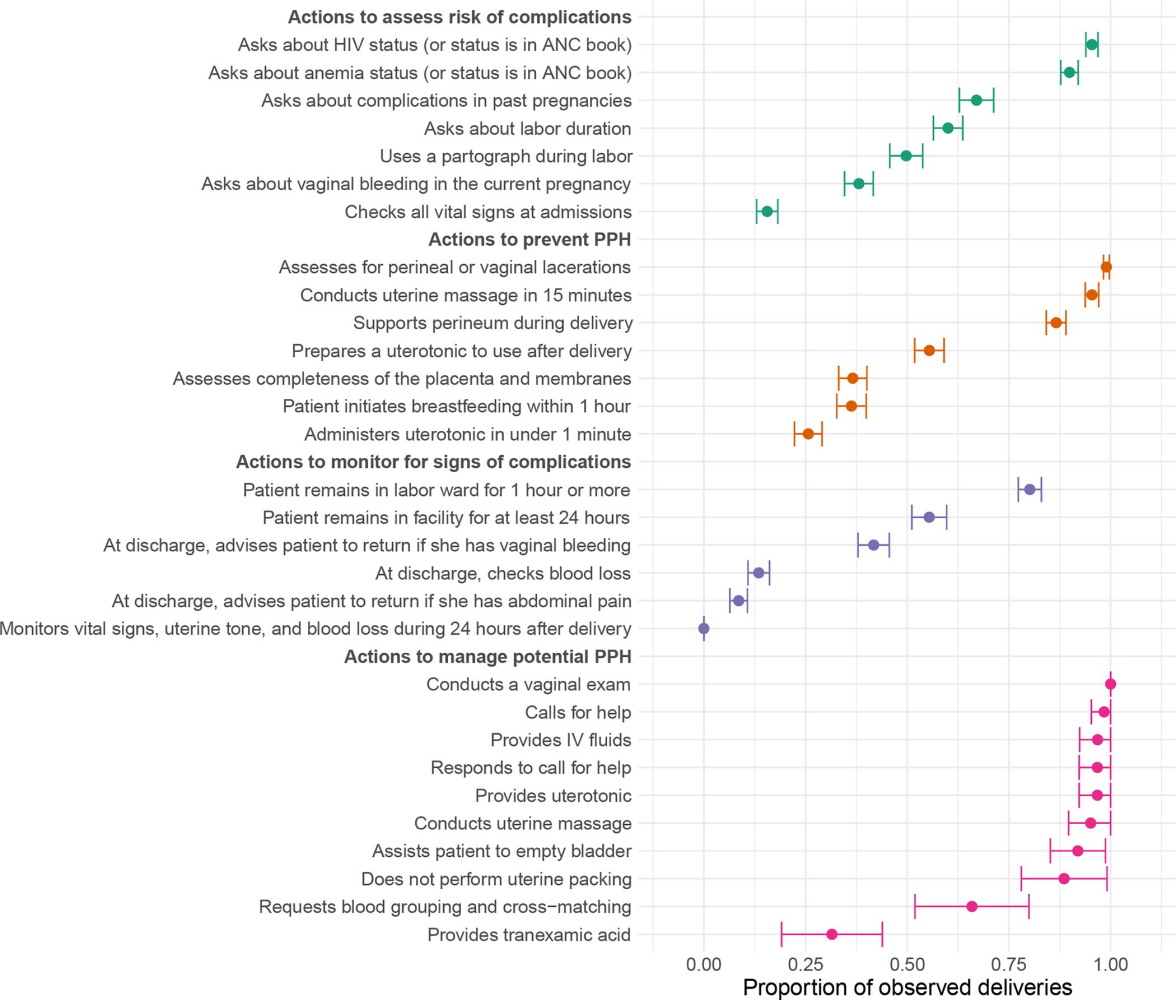
Adherence to guidelines for postpartum hemorrhage prevention, identification, and management. Notes: This figure shows the estimated proportion of observed deliveries in which each listed action was completed with 95% confidence intervals. Colors correspond to different aspects of quality care: Assessing risk of PPH, preventing PPH, monitoring for signs of PPH, and managing suspected PPH.

Adherence to guidelines varied across the categories of risk assessment, prevention, monitoring, and management (Fig 2). Providers adhered to guidelines for risk assessment in 7% (95% CI: 5% - 10%) of deliveries. While providers frequently asked patients about their HIV and anemia status (or this information was available in patients’ antenatal care books), assessment of other risk factors was limited. For example, providers asked about complications in past pregnancies in only 67% (95% CI: 63% - 71%) of deliveries (among women with past pregnancies) and checked all vital signs during the initial exam in only 16% (95% CI: 13% - 18%) of deliveries.

Providers completed all recommended actions for preventive care in 3% (95% CI: 1% - 4%) of deliveries. Providers almost always supported the perineum during delivery, assessed patients for perineal or vaginal lacerations following delivery, administered a prophylactic uterotonic following delivery, and performed uterine massage within 15 minutes of delivery (all over 87%). However, assessment of completeness of the placenta and membranes was less common (37%, 95% CI: 33% - 40%), and prophylactic uterotonics were administered in the recommended timeframe of 1 minute [22] in only 26% (95% CI: 22% - 29%) of deliveries.

Providers did not adhere to guidelines for postpartum monitoring, including assessment of vital signs, uterine tone, and blood loss during the 24 hours following delivery, in any of the observed deliveries. While 80% of patients (95% CI: 77% - 83%) remained in the labor ward for the recommended 1 hour or more and 55% remained in the hospital for at least 24 hours (95% CI: 51% - 60%), there were almost no assessments of vital signs, uterine tone, or blood loss during this period (Table 3). At the time of discharge, few patients were advised to return to a health facility in the case of bleeding (42%, 95% CI: 38% - 46%) or abdominal pain (9%, 95% CI: 6% - 11%).

**Table 3:** Total times patients were monitored, by amount of time remaining in facility post-delivery.

Time patient remained in facility after delivery	Number of patients	# of times patients should be monitored	Average number of times patients were monitored patients in total between delivery and discharge				
			Blood pressure (95% CI)	Pulse (95% CI)	Temperature (95% CI)	Uterine tone (95% CI)	Blood loss (95% CI)
> = 4 hours and <8 hours	3	At least 6	0.00	0.00	0.00	0.00	0.33 (0.00–0.68)
> = 8 hours and <12 hours	27	At least 7	0.50 (0.15–0.85)	0.50 (0.15–0.85)	0.17 (0.01–0.32)	0.11 (0.00–0.23)	0.33 (0.00–0.68)
> = 12 hours and <16 hours	45	At least 8	0.71 (0.43–1.00)	0.71 (0.43–1.00)	0.09 (0.00–0.18)	0.20 (0.03–0.37)	0.37 (0.10–0.64)
> = 16 hours and <20 hours	87	At least 9	0.74 (0.58–0.91)	0.74 (0.58–0.91)	0.23 (0.12–0.34)	0.16 (0.08–0.24)	0.26 (0.16–0.36)
> = 20 hours and <24 hours	85	At least 10	0.69 (0.52–0.85)	0.67 (0.51–0.84)	0.27 (0.15–0.38)	0.16 (0.05–0.28)	0.31 (0.19–0.44)
> = 24 hours	307	At least 11	1.13 (0.98–1.28)	1.08 (0.94–1.22)	0.48 (0.38–0.58)	0.19 (0.14–0.24)	0.46 (0.38–0.55)

Notes: Table shows the average total number of times patients were monitored between the time when they delivered and the time when they were discharged from the health facility, in comparison to recommendations from the World Health Organization. The first column indicates the amount of time the patient remained in the health facility. The second column indicates the number of patients in this category in our sample. The third column indicates the number of times patients should be monitored if they remain in the health facility for this amount of time, based on WHO recommendations. The remaining columns show the mean and 95% confidence intervals for the total number of times patients are monitored.

Adherence to clinical guidelines for PPH management was generally high. Providers called for help, responded to a call for help, conducted a vaginal exam, provided IV fluids, provided therapeutic uterotonics, conducted uterine massage, and assisted patients to empty their bladder in 92% of cases or more (95% CI: 85% -99%). Two exceptions were provision of tranexamic acid (31% of cases, 95% CI: 19% -44%), which was a recent addition to the WHO clinical guidelines at the time of data collection, and request for type and cross-match of blood (66% of cases, 95% CI: 52% -80%). Providers completed all recommended actions in 23% (95% CI: 6% - 40%) of cases.

### Timeliness of PPH care

**Fig 3:**
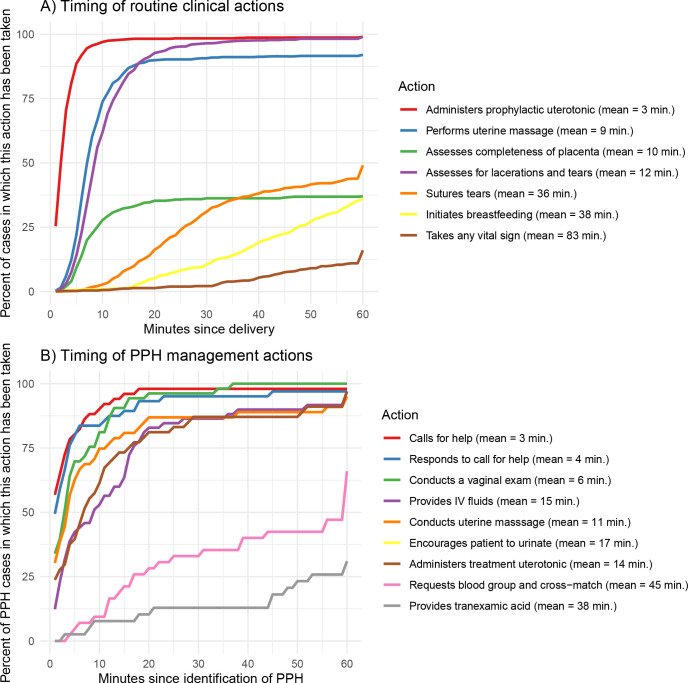
Timeliness of routine care and PPH management. Notes: This figure shows the cumulative distribution of the completion of different clinical actions over time. Panel A shows the timing of routine actions, measured in minutes since the delivery of the final newborn. Panel B shows the timing of actions related to PPH management, measured in minutes since the identification of PPH. Actions taking place more than 60 minutes after the delivery (for Panel A) or more than 60 minutes after PPH identification (for Panel B) are depicted as occurring at 60 minutes. The legend indicates the mean time when the action occurred, even if it occurred beyond the 60-minute interval shown on the figure. The mean timing for initiation of breastfeeding only includes patients who breastfed within 60 minutes of delivery. The mean time to “any vital sign” includes the time from delivery to the first time blood pressure, pulse, or temperature were taken, among those patients for whom any vital sign was taken while they remained in the labor & delivery ward.

Fig 3 shows the average timing and cumulative distribution of timing of time-sensitive clinical actions following delivery (Panel A) and following the identification of PPH (Panel B). Following delivery, administration of prophylactic uterotonics, uterine massage, assessment of the placenta, and assessment for lacerations and tears were all performed within 15 minutes on average. Other actions were more widely spread over the full first hour after delivery: for example, suturing was performed within 15 minutes of delivery in 9% of deliveries and within 60 minutes of delivery in 44% of deliveries. When obtained, vital signs were usually measured more than 30 minutes after delivery.

Typically, after identifying a case of PPH. providers called for help immediately (3 minutes on average). In many cases critical steps in PPH care were rapidly performed although, at times, 15 minutes or more passed before critical actions were performed. For example, treatment uterotonics were most commonly administered between 5 and 15 minutes after the identification of PPH, but 24% of patients did not receive treatment uterotonics until more than 15 minutes after PPH had been identified. In 32% of observed cases, requests for blood-type and cross-matching occurred more than 30 minutes after PPH was first identified (and in 33% of observed cases, these requests were not made at all).

### Sensitivity analyses

We estimated lower and upper bound estimates for the prevalence of suspected PPH of 8.3% (95% CI: 6.5% - 10.1%) and 9.2% (6.8% -11.6%).

Results from other sensitivity analyses are shown in S2 and S3 Tables. If missing actions were assumed to be not completed, then the estimated guideline adherence was lower than in the main analysis for actions with missing data. The most affected actions were partograph use (shift from 50% to 38%), uterine massage within 15 minutes of delivery (95% to 76%), not performing uterine packing during PPH management (89% to 50%) and requesting blood type and cross-matching during PPH management (66% to 47%). We found minimal changes in results with other sensitivity analyses.

## Discussion

This study identified significant gaps in adherence to guidelines for PPH care in three referral hospitals in Kenya. First, there was low coverage of key clinical actions to prevent PPH such as administration of prophylactic uterotonics within one minute of delivery and assessment of the placenta. Second, providers did not adhere to guidelines for monitoring patients for early signs of complications in any of the observed deliveries. Finally, when providers did adhere to guidelines, they did not always do so in a timely manner. In some cases, substantial delays were observed in the repair of tears and in obtaining vital signs after delivery and in basic interventions to manage PPH such as vaginal exams and uterine massage.

The observed gaps in quality of care may have important implications for morbidity and mortality from PPH. Timely administration of prophylactic uterotonics and assessment of the placenta and membranes are important for reducing the risk of PPH [3]. Many cases of PPH cannot be easily predicted based on patient characteristics [34] so monitoring of vital signs, blood loss, and uterine tone is needed to ensure early identification and timely action is needed so that complications can be managed before they become severe [35]. Tranexamic acid has been proven to be an effective treatment for PPH, so the low use we observed in this study is concerning [25].

Our findings are particularly concerning since the study hospitals serve as referral and teaching hospitals. Together, they perform over 30,000 deliveries annually. Moreover, the quality of care in these hospitals serves as a reference for providers who train there and go on to practice throughout the country. As researchers and policymakers consider ways to redesign health systems so that more patients deliver in hospitals or other advanced care health facilities [36], it will be important to address gaps in the quality of care at this level of the health system.

Our findings are consistent with a growing literature documenting gaps in maternity care quality in LMICs. This literature demonstrates that gaps in preventive care actions are common. For example, a 2015 study in six sub-Saharan African countries found that uterotonics were administered within 1 minute of delivery in 52% of observed deliveries [6]. While there has been less research on later postpartum monitoring, the existing evidence points to low rates of vital sign monitoring and blood less assessment [37]. Maternal death reviews from a variety of settings have identified insufficient monitoring as an important factor driving mortality [38].

Few prior studies have reported the timeliness of PPH care, an example of the “third delay” in maternity care [39]. One study in Benin, Ecuador, Jamaica, and Rwanda estimated that it took 52 to 57 minutes (25^th^-75^th^ percentile range) from PPH diagnosis to administration of therapeutic uterotonics [12], a much longer delay than in our sample. Our study contributes to the literature by describing the distribution of timing of several time-sensitive clinical actions following delivery and PPH identification. For example, we found significant delays in the repair of tears; this might have been driven by the fact sutures were generally not available bedside, so providers had to retrieve them.

Our study has several limitations. First, our findings could be influenced by Hawthorne effects, whereby providers adjust their behavior in the presence of observers. However, if this were a major concern, it would have led us to overestimate quality of care. Second, observers might not have captured all actions completed by health care providers, leading to an underestimate of adherence. We aimed to minimize this risk during observer training and piloting of research instruments. Notably, for some actions, such as the use of uterotonics, we observed very high rates of adherence. Many of the actions for which we observed low rates of adherence (e.g., blood pressure measurement) would have been easily observable. Third, while our study is focused on vaginal deliveries, many cases of PPH develop after caesarean sections. Additionally, we did not observe PPH cases that occur after women leave the hospital. Further research is needed to understand these cases. Fourth, there was a strike during our data collection period in one hospital. This may have decreased the quality of care; strikes have been shown to be associated with poor health outcomes [40]. However, health worker strikes are common in Kenya: for example, one study identified 32 health worker strikes from 1995–2014 [40]., Therefore, many patients experience the quality of care we observed during this period. Finally, our study could be biased if missing data were not missing completely at random. We examined the robustness of our findings to different assumptions about missing data, and our main findings did not change.

This study raises questions about the reasons for low quality PPH care. There are a number of factors that may play a role. First, there may be a need for improvements in the availability of supplies and equipment related to PPH care. While all of the study hospitals were supplied with oxytocin at the time of data collection, we observed blood shortages as well as stock-outs of tranexamic acid. In addition, the hospitals had very few blood pressure cuffs relative to the volume of patients they manage. Second, there may be need for improvements in staff availability or allocation in these high-volume hospitals; this is an important area for future research. However, supply shortages and inadequate staffing alone are unlikely to explain the observed gaps in quality. There is growing evidence of important gaps in health care provider knowledge of clinical protocols for maternity care, and in gaps between provider knowledge and actual practice (“know-do gaps”) [41]. Know-do gaps may be explained by a range of factors such as health facility norms, burnout or lack of motivation among health care providers, lack of accountability, or insufficient supportive supervision [41–43].

A critical next step is to develop and test approaches to improve PPH care. In the Kenyan setting, recent policy efforts have increased access to maternity care in health facilities, but it is critical to also focus on the quality of that care [15]. There is a need to address gaps in blood availability and ensure facilities are sufficiently supplied to prevent and manage PPH cases. In addition, our findings point to a need to improve emergency preparedness and timely clinical decision-making. Adoption of simulation training [44], early warning systems [45], and other quality improvement interventions [46] should be considered. The implementation of PPH care bundles, incorporating not only recommended clinical actions but also critical supporting elements such as high quality teamwork and communication, should also be considered [47]. Future research could attempt to explain why know-do gaps occur and how to close them.

We found significant gaps in adherence to clinical guidelines for PPH care in referral hospitals in Kenya. Global efforts to improve the quality of delivery care should include a focus on preventive care, postpartum monitoring, and timeliness.

## Supporting information

S1 Table Quality of care indicator sources and relevant samples. (DOCX)

S2 Table Results from sensitivity analyses: Missing data imputation. (DOCX)

S3 Table Postpartum monitoring between delivery and discharge from the health facility. (DOCX)

S4 Table Data description. (DOCX)

S1 Text Supplementary methods. (DOCX)

S2 Text PLOS GPH inclusivity questionnaire. (DOCX)

S1 Data Data. (DTA)
