# Supplementary material for: Quality of care for postpartum hemorrhage: A direct observation study in referral hospitals in Kenya
Source: PLOS Glob Public Health. 2023 Mar 2;3(3):e0001670. doi: 10.1371/journal.pgph.0001670 (PMC10022124; doi:10.1371/journal.pgph.0001670)
Supplement: S1 Table — (DOCX) [file pgph.0001670.s001.docx]

S1 Table: Quality of care indicator sources and relevant samples

| Indicator | Tripathi et al [4] | MCHIP [5] | Kenyan Guidelines [6] | WHO Standards for Maternity Care [7] | WHO Integrated Management guidelines [8] | WHO Postpartum Hemorrhage guidelines [9], [10] | Included in sample |
| --- | --- | --- | --- | --- | --- | --- | --- |
| 1. Actions to assess risk of complications | | | | | | | |
| (1) Checks woman’s HIV status (checks chart or asks woman) and/or offers woman an HIV test during initial exam | Item 1 | No | Page 173 | Page 25 | D2 | No | *All patients observed during initial exam* |
| (2) Checks woman’s anemia status (checks chart or asks woman) and/or offers woman a hemoglobin test during initial exam | No | No | Page 173 | Page 25 | D2 | No | *All patients observed during initial exam* |
| (3) Asks woman about history of complications during past pregnancies during initial exam | No | Page 19, Figure 5.2 | Page 173 | Page 25 | D2 | No | *All patients observed during initial exam* |
| (4) Asks the patient how long she has been in labor for during initial exam | No | No | Page 173 | No | D2 | No | *All patients observed during initial exam* |
| (5) Uses a partograph during labor | Item 10 | Page 21,  Table 5.5 | Page 183 | Page 26 | D3 | No | *All patients observed during initial exam* |
| (6) Asks whether woman has experienced vaginal bleeding during this pregnancy during initial exam | Item 3 | Page 19, Figure 5.2 | Page 173 | Page 25 | D5 | No | *All patients observed during initial exam* |
| (7) Takes vital signs (temperature, blood pressure, and pulse) during initial exam | Items 4-5 (Temperature not included) | Page 19, Figure 5.2 (Blood pressure not included) | Page 173 | Page 26 | D2 | No | *All patients observed during initial exam* |
| 1. Actions to prevent PPH | | | | | | | |
| (8) Assesses for perineal and vaginal lacerations | Item 14 | Page 19, Figure 5.3 | Page 181 | No | D13 | No | *All patients observed during delivery* |
| (9) Palpates uterus within 15 minutes of delivery | Item 18 | Page 19, Figure 5.3 | Page 180 | No | D13 | Page 4 | *All patients observed during delivery* |
| (10) Supports perineum during delivery | No | Page 19, Figure 5.3 | Page 177 | No | D11 | No | *All patients observed during delivery* |
| (11) Prepares uterotonic to use for active management of the third stage of labor | Item 9 | No | ? | No | Unclear. Listed in L3, but not clear if recommendation is to prepare in advance. | No | *All patients observed during delivery* |
| (12) Assists patients to initiate breastfeeding within 1 hour after delivery | Item 20 | Page 22, Figure 5.6 | Page 177 | Pages 27 and 28 | K2 | No | *All patients observed during delivery and for 1 hour afterwards, excluding those whose delivery ended in a stillbirth* |
| (13) Assesses completeness of the placenta and membranes | Item 13 | Page 19, Figure 5.3 | Page 181 | No | D12-D13 | No | *All patients observed during delivery* |
| (14) Administers uterotonic within 1 minute of delivery | Item 12 | Page 22, Figure 5.5 | Page 179 | Page 26 | D11 | Page 5 | *All patients observed during delivery* |
| 1. Actions to monitor for signs of complications | | | | | | | |
| (15) Patient remains in labor ward for 1 hour after delivery | No | No | Page 182 | No | D13 | No | *All patients observed during delivery and for 1 hour afterwards* |
| (16) Patient remains in the facility for at least 24 hours after delivery | No | No | ? | Page 28 | D20 | No | *All patients observed through discharge from the facility* |
| (17) At discharge, discusses maternal danger signs: severe abdominal pain | No | No | Page 247 | Pages 27, 46, and 54 (does not list specific danger signs) | D28 | No | *All patients observed through discharge from the facility* |
| (18) At discharge, assesses maternal blood loss | No | No | Page 247 | No | D28 | No | *All patients observed through discharge from the facility* |
| (19) At discharge, discusses maternal danger signs: bleeding | No | No | Page 247 | Pages 27, 46, and 54 (does not list specific danger signs) | D28 | No | *All patients observed through discharge from the facility* |
| (20) Follows guidelines for taking vital signs during 24 hours after delivery |  |  |  |  |  | No |  |
| - Takes vital signs within 15 minutes after birth (blood pressure, pulse, temperature) | Item 19 | Page 19, Figure 5.3 | Yes | Page 25 (does not specify frequency or timing) | D19 | No | *All patients observed during delivery* |
| - Takes vital signs 3 times from 15 minutes to 1 hour after delivery | No | No | Page 182 (with specified frequency and timing) | Pages 26 and 28 (does not specify frequency or timing) | D20 (with specified frequency and timing) | No | *All patients observed during delivery and for 1 hour afterwards* |
| - Takes vital signs hourly from hours 1-4 after delivery | No | No | Page 244 (does not specify frequency or timing) | Page 26 and 28 (does not specify frequency or timing) | D20 (with specified frequency and timing) | No | *All patients observed during delivery and for 4 hours afterwards* |
| - Takes vital signs every 4 hours from hours 4 to 24 after delivery | No | No | Page 244 (does not specify frequency or timing) | Page 26 and 28 (does not specify frequency or timing) | D20 (with specified frequency and timing) | No | *All patients observed during delivery and for 24 hours afterwards* |
| (21) Follows guidelines for assessing uterine tone during 24 hours after delivery |  |  |  |  |  |  |  |
| - Assesses uterine tone 3 times from 15 minutes to 1 hour after delivery | No | No | Page 244 (does not specify frequency or timing) | No | D20 (with specified frequency and timing) | No | *All patients observed during delivery and for 1 hour afterwards* |
| - Assesses uterine tone hourly from hours 1-4 after delivery | No | No | Page 244 (does not specify frequency or timing) | No | D20 (with specified frequency and timing) | No | *All patients observed during delivery and for 4 hours afterwards* |
| - Assesses uterine tone every 4 hours from hours 4 to 24 after delivery | No | No | Page 244 (does not specify frequency or timing) | No | D20 (with specified frequency and timing) | No | *All patients observed during delivery and for 24 hours afterwards* |
| (22) Follows guidelines for assessing blood loss during 24 hours after delivery |  |  |  |  |  |  |  |
| - Assesses blood loss 3 times from 15 minutes to 1 hour after delivery | No | No | Page 182 (with specified frequency and timing) | Page 28 (does not specify frequency, timing, or method) | D20 (with specified frequency, but not method) | No | *All patients observed during delivery and for 1 hour afterwards* |
| - Assesses blood loss hourly from hours 1-4 after delivery | No | No | Page 244 (does not specify frequency, timing, or method) | Page 28 (does not specify frequency, timing, or method) | D20 (with specified frequency and timing) | No | *All patients observed during delivery and for 4 hours afterwards* |
| - Assesses blood loss every 4 hours from hours 4 to 24 after delivery | No | No | Page 244 (does not specify frequency, timing, or method) | Page 28 (does not specify frequency, timing, or method) | D20 (with specified frequency and timing) | No | *All patients observed during delivery and for 24 hours afterwards* |
| 1. Management of suspected postpartum hemorrhage | | | | | | | |
| (23) Conducts a vaginal exam | No | No | Page 259 | No | Yes | No | *All patients observed receiving care for suspected PPH* |
| (24) Calls for help | No | No | Page 259 | No | B5 | No | *All patients observed receiving care for suspected PPH* |
| (25) Provides IV fluids | No | No | No | No | B5 | Page 6 | *All patients observed receiving care for suspected PPH* |
| (26) Another provider responds to the call for help | No | No | No | No | No | No | *All patients observed receiving care for suspected PPH* |
| (27) Administers uterotonics (oxytocin IM) | No | Page 25,  Table 5.8 | Page 259 | Page 30 | Yes | Page 6 | *All patients observed receiving care for suspected PPH* |
| (28) Continuous uterine massage | No | Page 25,  Table 5.8 | Page 259 | No | B5 | Page 6 | *All patients observed receiving care for suspected PPH* |
| (29) Assists patient to empty her bladder | No | No | Page 259 | No | B5 | No | *All patients observed receiving care for suspected PPH* |
| (30) Does not conduct uterine packing | No | No | Page 259 | No | No | Page 6 | *All patients observed receiving care for suspected PPH* |
| (31) Requests blood grouping and cross-matching | No | No | Page 259 | No | No | No | *All patients observed receiving care for suspected PPH* |
| (32) Provides tranexamic acid | No | No | ? | No | No | Page 6 | *All patients observed receiving care for suspected PPH* |
