# Supplementary material for: Quality of care for postpartum hemorrhage: A direct observation study in referral hospitals in Kenya
Source: PLOS Glob Public Health. 2023 Mar 2;3(3):e0001670. doi: 10.1371/journal.pgph.0001670 (PMC10022124; doi:10.1371/journal.pgph.0001670)
Supplement: S2 Table — (DOCX) [file pgph.0001670.s002.docx]

S2 Table. Results from sensitivity analysis: missing data imputation

| **Action** | **Percent missing** | **Missing data excluded (main analysis)** | | **Missing data assumed to be 0, if section observed** | | **Multiple imputation** | | **Time assumed to be within recommended period, if timing not observed** | |
| --- | --- | --- | --- | --- | --- | --- | --- | --- | --- |
|  |  | **Mean**  **(95% CI)** | **N** | **Mean** | **N** | **Mean** | **N** | **Mean** | **N** |
| **Actions to assess risk of complications** | | | | | | | | | |
| Asks about HIV status (or status is in ANC book) | <1% | 0.95  (0.94-0.97) | 760 | 0.95  (0.93, 0.96) | 767 | 0.95  (0.94, 0.97) | 767 | - | - |
| Asks about anemia status (or status is in ANC book) | <1% | 0.90  (0.88-0.92) | 761 | 0.89  (0.87, 0.91) | 767 | 0.90  (0.88, 0.92) | 767 | - | - |
| Asks about complications in past pregnancies | 4% | 0.67  (0.63-0.71) | 476 | 0.64  (0.60-0.68) | 498 | 0.68  (0.64, 0.73) | 498 | - | - |
| Asks about labor duration | 7% | 0.60  (0.56-0.64) | 708 | 0.55  (0.52, 0.59) | 767 | 0.60  (0.56-0.63) | 767 | - | - |
| Uses a partograph during labor | 22% | 0.50  (0.46-0.54) | 591 | 0.38  (0.34, 0.42) | 767 | 0.48  (0.43, 0.52) | 767 | - | - |
| Asks about vaginal bleeding in the current pregnancy | 5% | 0.38  (0.35-0.42) | 727 | 0.36  (0.33, 0.4) | 767 | 0.38  (0.35, 0.42) | 767 | - | - |
| Checks all vital signs at admissions | 3% | 0.16  (0.13-0.18) | 740 | 0.15  (0.12, 0.18) | 767 | 0.16  (0.13, 0.18) | 767 | - | - |
| **Actions to prevent PPH** | | | | | | | | | |
| Assesses for perineal or vaginal lacerations | 1% | 0.99  (0.98-1.00) | 762 | 0.98  (0.98, 0.99) | 766 | 0.99  (0.99-1.00) | 766 | - | - |
| Conducts uterine massage in 15 minutes | 21% | 0.95  (0.94-0.97) | 610 | 0.76  (0.73, 0.79) | 766 | 0.96  (0.94-0.97) | 766 | 0.96  (0.94-0.97) | 696 |
| Supports perineum | 1% | 0.87  (0.84-0.89) | 761 | 0.86  (0.84, 0.88) | 766 | 0.87  (0.84-0.89) | 766 | - | - |
| Prepares a uterotonic to use after delivery | 6% | 0.55  (0.52-0.59) | 727 | 0.53  (0.49, 0.56) | 766 | 0.55  (0.52-0.59) | 766 | - | - |
| Patient initiates breastfeeding within 1 hour | 12% | 0.36  (0.33-.40) | 678 | 0.33  (0.29, 0.37) | 745 | 0.39  (0.35-0.42) | 745 | - | - |
| Assesses completeness of the placenta and membranes | 4% | 0.37  (0.33-0.40) | 743 | 0.36  (0.32, 0.39) | 766 | 0.36  (0.33-0.40) | 766 | - | - |
| Administers uterotonic in under 1 minute | 17% | 0.26  (0.22-0.29) | 639 | 0.21  (0.18, 0.25) | 766 | 0.25  (0.22-0.28) | 766 | 0.37  (0.33-0.40) | 764 |
| **Actions to monitor for signs of complications** | | | | | | | | | |
| Patient remains in labor ward for 1 hour or more | 0% | 0.80  (0.77-0.83) | 760 | 0.80  (0.77-0.83) | 760 | 0.80  (0.77-0.83) | 760 | - | - |
| Patient remains in facility for at least 24 hours | 22% | 0.55  (0.51-0.60) | 518 | 0.43  (0.39, 0.47) | 665 | 0.56  (0.52, 0.61) | 665 | - | - |
| At discharge, advises patient to return if she has abdominal pain | 5% | 0.09  (0.06-0.11) | 635 | 0.08  (0.06, 0.10) | 665 | 0.11  (0.09-0.14) | 665 | - | - |
| At discharge, checks blood loss | 8% | 0.13  (0.11-0.16) | 609 | 0.13  (0.10, 0.16) | 665 | 0.15  (0.12-0.17) | 665 | - | - |
| At discharge, advises patient to return if she has vaginal bleeding | 5% | 0.42  (0.38-0.46) | 635 | 0.40  (0.36, 0.44) | 665 | 0.44 (0.40-0.48) | 665 | - | - |
| Follows guidelines for taking vital signs during 24 hours after delivery | 0% | 0.00  (0.00-0.00) | 265 | 0.00  (0.00-0.00) | 287 | 0.00  (0.00-0.00) | 287 | 0.00  (0.00-0.00) | 287 |
| Follows guidelines for assessing uterine tone during 24 hours after delivery | 0% | 0.00  (0.00-0.00) | 284 | 0.00  (0.00-0.00) | 287 | 0.00  (0.00-0.00) | 287 | 0.00  (0.00-0.00) | 287 |
| Follows guidelines for assessing blood loss during 24 hours after delivery | 0% | 0.00  (0.00-0.00) | 277 | 0.00  (0.00-0.00) | 287 | 0.00  (0.00-0.00) | 287 | 0.00  (0.00-0.00) | 287 |
| **Actions to manage suspected PPH** | | | | | | | | | |
| Conducts a vaginal exam | 0% | 1.00  (1.00-1.00) | 62 | 1.00  (1.00-1.00) | 62 | 1.00  (1.00-1.00) | 62 | - | - |
| Calls for help | 0% | .98  (0.95-1.00) | 62 | .98  (0.95-1.00) | 62 | .98  (0.95-1.00) | 62 | - | - |
| Provides IV fluids | 0% | .97  (0.92-1.00) | 62 | .97  (0.92-1.00) | 62 | .97  (0.92-1.00) | 62 | - | - |
| Responds to call for help | 2% | .97  (0.92-1.00) | 61 | 0.95  (0.90, 1.00) | 62 | 0.95  (0.90, 1.00) | 62 | - | - |
| Provides uterotonic | 0% | .97  (0.92-1.00) | 62 | .97  (0.92-1.00) | 62 | .97  (0.92-1.00) | 62 | - | - |
| Conducts uterine massage | 2% | .95  (0.90-1.00) | 61 | 0.94  (0.87, 1.00) | 62 | 0.95  (0.90-1.00) | 62 | - | - |
| Assists patient to empty bladder | 0% | .92  (0.85-0.99) | 62 | .92  (0.85-0.99) | 62 | 0.92  (0.85-0.99) | 62 | - | - |
| Does not perform uterine packing | 44% | 0.89  (0.78-0.99) | 35 | 0.50  (0.33, 0.67) | 62 | 0.92 (0.70-1.00) | 62 | - | - |
| Requests blood grouping and cross-matching | 29% | 0.66  (0.52-0.80) | 44 | 0.47  (0.32, 0.62) | 62 | 0.66  (0.54-0.79) | 62 | - | - |
| Provides tranexamic acid | 13% | 0.31  (0.19-0.44) | 54 | 0.27  (0.16, 0.39) | 62 | 0.31  (0.19-0.44) | 62 | - | - |

^1^The “percentage missing” is presented as a percent of the cases for which the relevant delivery phase was observed. If the action should take place during admissions, then the denominator for calculating the “percentage missing” is 767 (except for asking about past complications, which is not relevant for patients going through their first delivery so has a denominator of 498). If the action should take place during the delivery or the first 15 minutes after the delivery, then the denominator is 766. If the action should take place from 15 minutes to 60 minutes after delivery, then the denominator is 760 (except for breastfeeding, which is only relevant for patients with a life birth so has a denominator of 745). If the action should take place during the later postpartum period (through 24 hours after delivery), then the denominator is 287 (because this is the number of patients who were observed through the later postpartum period and remained in the facility for at least 24 hours). If the action should take place at discharge from the health facility, then the denominator is 665. Finally, if the action should take place during PPH management, then the denominator is 62.
