# Supplementary material for: Quality of care for postpartum hemorrhage: A direct observation study in referral hospitals in Kenya
Source: PLOS Glob Public Health. 2023 Mar 2;3(3):e0001670. doi: 10.1371/journal.pgph.0001670 (PMC10022124; doi:10.1371/journal.pgph.0001670)
Supplement: S3 Table — (DOCX) [file pgph.0001670.s003.docx]

S3 Table: Postpartum monitoring between delivery and discharge from the health facility

| Assessment | Number of times assessment was conducted during this time interval | | | Assessment never conducted  (% of observations) | N |
| --- | --- | --- | --- | --- | --- |
|  | Mean | Standard deviation | Range |  |  |
| *0-15 minutes after delivery* | | | | |  |
| Blood pressure | 0.01 | 0.12 | 0-2 | 99% | 702 |
| Pulse | 0.01 | 0.12 | 0-2 | 99% | 704 |
| Temperature | 0.01 | 0.10 | 0-2 | 99% | 737 |
| Uterine tone | 0.75 | 0.44 | 0-2 | 26% | 762 |
| *16-60 minutes after delivery* | | | | |  |
| Blood pressure | 0.14 | 0.41 | 0-3 | 89% | 715 |
| Pulse | 0.13 | 0.38 | 0-2 | 89% | 714 |
| Temperature | 0.04 | 0.21 | 0-2 | 97% | 749 |
| Uterine tone | 0.04 | 0.20 | 0-1 | 96% | 733 |
| Blood loss | 0.04 | 0.19 | 0-1 | 96% | 751 |
| *1-4 hours after delivery* | | | | |  |
| Blood pressure | 0.27 | 0.55 | 0-6 | 77% | 593 |
| Pulse | 0.26 | 0.54 | 0-6 | 77% | 590 |
| Temperature | 0.07 | 0.29 | 0-3 | 94% | 626 |
| Uterine tone | 0.05 | 0.23 | 0-2 | 95% | 627 |
| Blood loss | 0.08 | 0.27 | 0-2 | 93% | 610 |
| *4-24 hours after delivery* | | | | |  |
| Blood pressure | 0.47 | 0.71 | 0-3 | 64% | 272 |
| Pulse | 0.46 | 0.71 | 0-3 | 65% | 269 |
| Temperature | 0.27 | 0.56 | 0-3 | 78% | 282 |
| Uterine tone | 0.06 | 0.23 | 0-1 | 95% | 284 |
| Blood loss | 0.24 | 0.50 | 0-3 | 79% | 277 |
| *At discharge from the facility* | | | | |  |
| Blood pressure | NA (binary) | NA (binary) | 0-1 | 90% | 699 |
| Pulse | NA (binary) | NA (binary) | 0-1 | 90% | 699 |
| Temperature | NA (binary) | NA (binary) | 0-1 | 89% | 699 |
| Blood loss | NA (binary) | NA (binary) | 0-1 | 85% | 592 |
