# Supplementary material for: Quality of care for postpartum hemorrhage: A direct observation study in referral hospitals in Kenya
Source: PLOS Glob Public Health. 2023 Mar 2;3(3):e0001670. doi: 10.1371/journal.pgph.0001670 (PMC10022124; doi:10.1371/journal.pgph.0001670)
Supplement: S4 Table — (DOCX) [file pgph.0001670.s004.docx]

S4 Table: Data Description

This file accompanies S1 Data.

S1 Data provides the dataset analyzed in this manuscript in Stata format. Health facility names and observation dates have been removed to protect the anonymity of the study participants.

The variables included are listed below.

|  | **Variable ename** | **Type** | **Numeric (yes or no)** | **Format** | **Variable label** |
| --- | --- | --- | --- | --- | --- |
| 1 | facility_id | float | 1 | %9.0g | Facility ID |
| 2 | patient_id | str7 | 0 | %9s | Patient ID |
| 3 | firstexam_observed | byte | 1 | %92.0g | Was Section 2 (initial exam) observed? |
| 4 | delivery_observed | float | 1 | %68.0g | Was Section 3 (delivery & immediate postpartum care) observed? |
| 5 | fourthstage_observed | float | 1 | %9.0g | Fourth stage of labor was observed |
| 6 | laterpostpartumcare_observed | float | 1 | %9.0g | Was Sections 5 (postpartum care after 1 hr) observed through 24 hr or discharge? |
| 7 | discharge_observed | float | 1 | %9.0g | Was Section 7 (patient discharge) observed? |
| 8 | pphcase | float | 1 | %9.0g | PPH case (with PPH form completed) |
| 9 | pphcase_notes | float | 1 | %9.0g | PPH case (includes cases identified based on observer notes) |
| 10 | ldp_sec1_q103a | byte | 1 | %21.0g | Q103: Reason for referral |
| 11 | ldp_sc2_q201a1 | byte | 1 | %44.0g | Q201: Asks about complications during previous pregnancies. |
| 12 | ldp_sc2_q201a2_1 | byte | 1 | %8.0g | Q201: Mother's response: Miscarriage |
| 13 | ldp_sc2_q201a2_2 | byte | 1 | %8.0g | Q201: Mother's response: Pre-eclampsia / eclampsia |
| 14 | ldp_sc2_q201a2_3 | byte | 1 | %8.0g | Q201: Mother's response: Bleeding during pregnancy |
| 15 | ldp_sc2_q201a2_4 | byte | 1 | %8.0g | Q201: Mother's response: Multiple birth |
| 16 | ldp_sc2_q201a2_5 | byte | 1 | %8.0g | Q201: Mother's response: Stillbirth |
| 17 | ldp_sc2_q201a2_6 | byte | 1 | %8.0g | Q201: Mother's response: PPH |
| 18 | ldp_sc2_q201a2_7 | byte | 1 | %8.0g | Q201: Mother's response: Other |
| 19 | ldp_sc2_q201a2_8 | byte | 1 | %8.0g | Q201: Mother's response: None |
| 20 | ldp_sc2_pastcaesarean | float | 1 | %21.0g | Past caesarean: combined from Q201 and Q202 |
| 21 | ldp_sc2_q204a1 | byte | 1 | %44.0g | Q204: Asks whether she has experienced vaginal bleeding during the current pregn |
| 22 | ldp_sc2_q209a1 | byte | 1 | %44.0g | Q209: Asks how long she has been in labour |
| 23 | ldp_sc2_q215a1 | byte | 1 | %44.0g | Q215: Takes temperature |
| 24 | ldp_sc2_q216a1 | byte | 1 | %44.0g | Q216: Takes pulse |
| 25 | ldp_sc2_q217a1 | byte | 1 | %44.0g | Q217: Takes blood pressure |
| 26 | ldp_sc2_allvitals | float | 1 | %9.0g | Q215-217: All vital signs taken during initial exam. |
| 27 | ldp_sc2_q225a2 | byte | 1 | %8.0g | Q225: What is the age of the mother? |
| 28 | ldp_sc2_q227a2 | byte | 1 | %8.0g | Q227: Gravidity |
| 29 | firstpregnancy | float | 1 | %9.0g | Is this her first pregnancy? (Based on gravidity from ANC book) |
| 30 | ldp_sc2_q228a2 | byte | 1 | %8.0g | Q228: Parity (first number = live births) |
| 31 | ldp_sc2_q228a3 | byte | 1 | %8.0g | Q228: Parity (second number = lost pregnancies before 28 weeks) |
| 32 | ldp_sc2_q230b2 | byte | 1 | %8.0g | Q230: How many antenatal care visits did the mother attend? |
| 33 | ldp_sc2_q234a1 | byte | 1 | %12.0g | Q234: Was the mother's haemoglobin measured during antenatal care? |
| 34 | ldp_sc2_q234a5 | float | 1 | %9.0g | Q234: Record the Hb level. |
| 35 | ldp_sc2_anemiatestorquestion | float | 1 | %10.0g | At least one of the following: the provider asked about anemia, the ANC book inc |
| 36 | ldp_sc2_q235a1 | byte | 1 | %12.0g | Q235: Did the mother receive iron and folate tablets during antenatal care? |
| 37 | ldp_sc2_HIVtestorquestion | float | 1 | %10.0g | At least one of the following: the provider asked about HIV status, the ANC book |
| 38 | ldp_sc2_q238 | byte | 1 | %20.0g | Q238: What is the HIV status recorded in the book? |
| 39 | ldp_sc2_q239a1 | byte | 1 | %12.0g | Q239: Was the mother treated for anaemia during antenatal care? |
| 40 | ldp_sc2_q240 | byte | 1 | %12.0g | Q240: Did the mother have antepartum haemorrhage (APH) during this pregnancy? |
| 41 | ldp_sc2_q245a1 | byte | 1 | %10.0g | Q245: Is a companion present with the mother? |
| 42 | ldp_sc2_q245a2_categories | long | 1 | %34.0g | Q245: Relationship of birth companion to mother |
| 43 | ldp_sec3_q303 | byte | 1 | %12.0g | Q303: Has prepared uterotonic to use for AMTSL. (If oxytocin or ergometrine, it |
| 44 | ldp_sec3_q315a1 | byte | 1 | %12.0g | Q315: As baby’s head is delivered, supports perineum. |
| 45 | ldp_sec3_q324a1 | byte | 1 | %12.0g | Q324: Administers uterotonic |
| 46 | ldp_sec3_lastbabytouterotonic | double | 1 | %10.0g | Time from birth of last baby to prophylactic uterotonics (minutes) |
| 47 | ldp_sec3_uterotonic_under1min | float | 1 | %9.0g | Uterotonic in under 1 minute (yes or no) |
| 48 | ldp_sec3_q327a1 | byte | 1 | %12.0g | Q327: Assesses completeness of the placenta and membranes by examining both the |
| 49 | ldp_sec3_lastbabytoplacenta | double | 1 | %10.0g | Time from birth of last baby to assessment of placenta (minutes) |
| 50 | ldp_sec3_q328a1 | byte | 1 | %12.0g | Q328: Performs uterine massage |
| 51 | ldp_sec3_lastbabytomassage | double | 1 | %10.0g | Time from birth of last baby to uterine massage (minutes) |
| 52 | ldp_sec3_babytomassage_under15 | float | 1 | %9.0g | Uterotonic in 15 minutes (yes or no) |
| 53 | ldp_sec3_q329a1 | byte | 1 | %12.0g | Q329: Assesses for perineal and vaginal lacerations |
| 54 | ldp_sec3_lastbabytotears | double | 1 | %10.0g | Time from birth of last baby to assessment of tears (minutes) |
| 55 | ldp_sec3_q345a1 | byte | 1 | %12.0g | Q345: Takes mother's blood pressure |
| 56 | ldp_sec3_q346a1 | byte | 1 | %12.0g | Q346: Takes mother's pulse |
| 57 | ldp_sec3_q347a1 | int | 1 | %12.0g | Q347: Takes mother's temperature |
| 58 | ldp_sec3_q385 | byte | 1 | %17.0g | Q385: Was the mother’s companion present with her during this stage? |
| 59 | time_babytoleftlabor | float | 1 | %9.0g | Time remaining in labor ward after delivery (hours) |
| 60 | time_babytoleftlabor_over1hr | float | 1 | %9.0g | Remained in labor ward for 60+ minutes after delivery |
| 61 | ldp_sec7_q708 | byte | 1 | %12.0g | Q708: Checks mother’s temperature |
| 62 | ldp_sec7_q709 | byte | 1 | %12.0g | Q709: Checks mother’s blood pressure |
| 63 | ldp_sec7_q710 | byte | 1 | %12.0g | Q710: Checks mother’s pulse |
| 64 | ldp_sec7_q711 | byte | 1 | %12.0g | Q711: Checks vaginal blood loss |
| 65 | ldp_sec7_q720a | byte | 1 | %12.0g | Q720a: Advises mother to return immediately if she has: vaginal bleeding. |
| 66 | ldp_sec7_q720g | byte | 1 | %12.0g | Q720g: Advises mother to return immediately if she has: epigastric pain / cramps |
| 67 | pph_sec6_callforhelp_yn | float | 1 | %9.0g | Q608: Was a call for help made? |
| 68 | pph_sec6_respondtocall_yn | float | 1 | %9.0g | Q611: Did any provider respond to the call for help? |
| 69 | pph_sec6_urination_yn | float | 1 | %9.0g | Q612: Did a provider ever encourage the patient to urinate after identification |
| 70 | pph_sec6_vaginalexam_yn | float | 1 | %9.0g | Q617: Did a provider ever conduct a vaginal exam after the identification of PPH |
| 71 | pph_sec6_q624 | byte | 1 | %10.0g | Q624: Requests blood grouping and cross-matching |
| 72 | pph_sec6_q630 | byte | 1 | %10.0g | Q6230: Places an IV |
| 73 | pph_sec6_ivfluid_yn | float | 1 | %9.0g | Did a provider ever provide IV fluid after the identification of PPH? |
| 74 | pph_sec6_uterinemassage_yn | float | 1 | %9.0g | Did a provider ever conduct a uterine massage after the identification of PPH? |
| 75 | pph_sec6_uterotonic_yn | float | 1 | %9.0g | Did a provider ever give a treatment uterotonic for PPH? |
| 76 | pph_sec6_tranexamic_yn | float | 1 | %9.0g | Did a provider ever provide tranexamic acid in response to PPH? |
| 77 | pph_sec6_q687 | byte | 1 | %10.0g | Q687: Performs uterine packing |
| 78 | pphcause_noneselected | float | 1 | %9.0g | Q699: PPH cause (recoded): none selected |
| 79 | pphcause_allthree | float | 1 | %9.0g | Q699: PPH cause (recoded): lacerations, atony, and retained products of concepti |
| 80 | pphcause_lacerationsrpoc | float | 1 | %9.0g | Q699: PPH cause (recoded): lacerations and retained products of conception |
| 81 | pphcause_atonyrpoc | float | 1 | %9.0g | Q699: PPH cause (recoded): atony and retained products of conception |
| 82 | pphcause_atonylacerations | float | 1 | %9.0g | Q699: PPH cause (recoded): lacerations and atony |
| 83 | pphcause_lacerationonly | float | 1 | %9.0g | Q699: PPH cause (recoded): lacerations ONLY |
| 84 | pphcause_rpoconly | float | 1 | %9.0g | Q699: PPH cause (recoded): retained products ONLY |
| 85 | pphcause_atonyonly | float | 1 | %9.0g | Q699: PPH cause (recoded): atony ONLY |
| 86 | ldp_sec3_lastbabytosuture | double | 1 | %10.0g | Time from birth of last baby to suturing tears (minutes) |
| 87 | ldp_sec3_lastbabytobreastfeeding | double | 1 | %10.0g | Time from birth of last baby to breastfeeding initiation (minutes) |
| 88 | time_babytodischarge_24plus | float | 1 | %9.0g | 24 hours or more between delivery and discharge from facility |
| 89 | time_deliverytopphid | float | 1 | %9.0g | Time from delivery until identification of PPH (minutes) |
| 90 | time_pphid_to_callforhelp | float | 1 | %9.0g | Time from PPH identification until call for help |
| 91 | time_pphid_to_response | float | 1 | %9.0g | Time from PPH identification until a provider responds to a call for help |
| 92 | time_pphid_to_vaginalexam | float | 1 | %9.0g | Time from PPH identification until a vaginal exam is performed |
| 93 | time_pphid_to_ivfluid | float | 1 | %9.0g | Time from PPH identification until IV fluid is first administered |
| 94 | time_pphid_to_massage | float | 1 | %9.0g | Time from PPH identifcation until uterine massage |
| 95 | time_pphid_to_urination | float | 1 | %9.0g | Time from PPH identification until provider encourages patient to urinate |
| 96 | time_pphid_to_uterotonic | float | 1 | %9.0g | Time from PPH identification until treatment uterotonic is administered |
| 97 | time_pphid_to_crossmatch | float | 1 | %9.0g | Time from PPH identification until blood grouping and cross-matching is done |
| 98 | time_pphid_to_tranexamic | float | 1 | %9.0g | Time from PPH identification until tranexamic acid is administered |
| 99 | partograph_true | float | 1 | %9.0g | Was the partograph filled out during labor? |
| 100 | ldp_sec3_lastbabytobp | double | 1 | %10.0g | Time from last baby to first blood pressure measurement |
| 101 | ldp_sec3_lastbabytopulse | double | 1 | %10.0g | Time from last baby to first pulse measurement |
| 102 | ldp_sec3_lastbabytotemp | double | 1 | %10.0g | Time from last baby to first temperature measurement |
| 103 | allvitals_first15 | float | 1 | %9.0g | Took blood pressure, pulse, and temperature within 15 minutes of delivery |
| 104 | allvitals_15to60 | float | 1 | %9.0g | Took blood pressure, pulse, and temperature at least once between 15 and 60 minu |
| 105 | bp_first15_binary | float | 1 | %9.0g | BP taken in first 15 minutes post-delivery (binary) |
| 106 | temp_first15_binary | float | 1 | %9.0g | Temperature taken in first 15 minutes post-delivery (binary) |
| 107 | pulse_first15_binary | float | 1 | %9.0g | Pulse taken in first 15 minutes post-delivery (binary) |
| 108 | tone_first15_binary | float | 1 | %9.0g | Uterine tone assessed in first 15 minutes post-delivery (binary) |
| 109 | bp_15to60_3x | float | 1 | %9.0g | BP taken 3 times from 15-60 minutes post-delivery |
| 110 | pulse_15to60_3x | float | 1 | %9.0g | Pulse taken 3 times from 15-60 minutes post-delivery |
| 111 | temp_15to60_3x | float | 1 | %9.0g | Temperature taken 3 times from 15-60 minutes post-delivery |
| 112 | blood_15to60_3x | float | 1 | %9.0g | Blood loss assessed 3 times from 15-60 minutes post-delivery |
| 113 | tone_15to60_3x | float | 1 | %9.0g | Uterine tone assessed 3 times from 15-60 minutes post-delivery |
| 114 | bp_hr1to4_3x | float | 1 | %9.0g | Blood pressure taken 3 times from 1 to 4 hours post-delivery |
| 115 | pulse_hr1to4_3x | float | 1 | %9.0g | Pulse taken 3 times from 1 to 4 hours post-delivery |
| 116 | temp_hr1to4_3x | float | 1 | %9.0g | Temperature taken 3 times from 1 to 4 hours post-delivery |
| 117 | blood_hr1to4_3x | float | 1 | %9.0g | Blood loss assessed 3 times from 1 to 4 hours post-delivery |
| 118 | tone_hr1to4_3x | float | 1 | %9.0g | Uterine tone assessed 3 times from 1 to 4 hours post-delivery |
| 119 | bp_hr4to24_4x | float | 1 | %9.0g | Blood pressure taken 4 times from 4 to 24 hours post-delivery |
| 120 | pulse_hr4to24_4x | float | 1 | %9.0g | Pulse taken 4 times from 4 to 24 hours post-delivery |
| 121 | temp_hr4to24_4x | float | 1 | %9.0g | Temperature taken 4 times from 4 to 24 hours post-delivery |
| 122 | blood_hr4to24_4x | float | 1 | %9.0g | Blood loss assessed 4 times from 4 to 24 hours post-delivery |
| 123 | tone_hr4to24_4x | float | 1 | %9.0g | Uterine tone assessed 4 times from 4 to 24 hours post-delivery |
| 124 | bp_laborward | float | 1 | %9.0g | BP taken while patient still in labor ward (yes or no) |
| 125 | temp_laborward | float | 1 | %9.0g | Temp taken while patient still in labor ward (yes or no) |
| 126 | pulse_laborward | float | 1 | %9.0g | Pulse taken while patient still in labor ward (yes or no) |
| 127 | anyvital_laborward | float | 1 | %9.0g | Any vital sign taken while patient still in labor ward (yes or no) |
| 128 | allvitals_hr1to4_3x | float | 1 | %9.0g | All vital signs taken 3 times from 1 to 4 hours post-delivery |
| 129 | allvitals_hr4to24_4x | float | 1 | %9.0g | All vital signs taken 4 times from 4 to 24 hours post-delivery |
| 130 | bp_totaltimes | float | 1 | %9.0g | Total times BP taken during observation |
| 131 | pulse_totaltimes | float | 1 | %9.0g | Total times pulse taken during observation |
| 132 | temp_totaltimes | float | 1 | %9.0g | Total times temperature taken during observation |
| 133 | blood_totaltimes | float | 1 | %9.0g | Total times blood loss assessed during observation |
| 134 | tone_totaltimes | float | 1 | %9.0g | Total times uterine tone assessed during observation |
| 135 | breastfeeding | float | 1 | %9.0g | Q349, with stillbirths taken out |
| 136 | admissions_student | float | 1 | %9.0g | Admissions assigned to student |
| 137 | delivery_student | float | 1 | %9.0g | Delivery assigned to student |
| 138 | hbunder11 | float | 1 | %9.0g | Hb under 11 during pregnancy |
| 139 | initialexam_all | float | 1 | %9.0g | Quality of care for PPH, initial exam: were all relevant steps completed? |
| 140 | firststage_all | float | 1 | %9.0g | Quality of care for PPH, first stage: were all relevant steps completed? |
| 141 | first15_all | float | 1 | %9.0g | Quality of care for PPH, delivery: were all relevant steps completed? |
| 142 | next45_all | float | 1 | %9.0g | Quality of care for PPH, 15min-60min: were all relevant steps taken? |
| 143 | all_laterpostpartumcare | float | 1 | %9.0g | Quality of care for PPH, later postpartum: were all relevant steps completed? |
| 144 | pphmanagement_all | float | 1 | %9.0g | Quality of care for PPH, PPH management: were all relevant steps completed? |
| 145 | time_in_facility | str24 | 0 | %24s | Total time patient remained in facility post-delivery |
