# Supplementary material for: Quality of care for postpartum hemorrhage: A direct observation study in referral hospitals in Kenya
Source: PLOS Glob Public Health. 2023 Mar 2;3(3):e0001670. doi: 10.1371/journal.pgph.0001670 (PMC10022124; doi:10.1371/journal.pgph.0001670)
Supplement: S1 Text — (DOCX) [file pgph.0001670.s005.docx]

**S1. Supplementary Methods**

*Bound estimation for the prevalence of suspected PPH*

The estimated rate of suspected PPH in the full study sample could be biased upwards because observers prioritized cases of suspected PPH for observation at times when the facility was too busy to observe all patients. It could also be biased downwards because some observations were censored (before patients were discharged or reached the 24-hour point) when observer shifts ended; if censored cases developed PPH after the observation team left the facility, this may not have been captured on the delivery observation form.

We therefore calculated an upper and lower bound on the rate of suspected PPH in the sample. The upper bound is the rate of PPH among completed delivery observations, i.e. cases that were observed from admissions through discharge from the facility. This is an upper bound because observations of PPH cases were more likely to be completed than observations of other (non-PPH) cases. The lower bound is the rate of PPH among all patients who were observed delivering. This is a lower bound because some of the censored patients may have developed PPH after the delivery observation ended (in cases when observations ended early due to the end of observer shifts). PPH cases could not be prioritized prior to delivery because PPH cannot be diagnosed until after delivery.

In our main analysis, we estimated the rate of suspected PPH among cases where the delivery was observed (75 out of 766 cases). This could be biased upwards if PPH deliveries were more likely to be observed than other deliveries, or it could be biased downwards if some patients were censored after their deliver prior to their development of PPH. We therefore also calculated upper and lower bound estimates. To estimate an upper bound, we estimated the rate of suspected PPH among the complete observations only. This represents an upper bound because, based on our study protocol, PPH cases were more likely to be completely observed than non-PPH cases. We estimated this bound as 10.5% (95% CI: 7.9%, 13.1%). To estimate a lower bound, we estimated the rate of suspected PPH among all patients who were ever observed for any portion of their labor, delivery, and postpartum care. This represents a lower bound because it includes censored patients (e.g. those who were observed for admission or delivery but not for postpartum care) in the denominator but assumes that they did not develop PPH, even though it is possible that they developed PPH during the postpartum period when they were not being observed. We estimated this bound as 9.3% (95% CI: 7.4%, 11.1%).

The fact that the upper and lower bounds are very close to each other suggests that few PPH cases were missed during the observation period, and the selective completion of PPH observations over other observations was not substantial enough to change the estimate by a lot.

*Missing data analysis*

In some cases, data were missing for one of four reasons: (a) a particular phase of the delivery was not observed for a particular patient (e.g. because the observation started after that phase had been completed, because observers were on a break during that phase, or because the observation ended before that phase ended), (b) an observer recorded that they “did not know” whether a particular clinical action had been completed (e.g. because they could not see it, or they were not sure what they were seeing), (c) an observer left a checklist item blank, or (d) an impossible response was included on the observation form (e.g. the times on the form suggested that the prophylactic uterotonic was given before the patient was admitted to the facility) and was therefore coded as missing.

For routine care indicators, data were missing in less than 5% of cases for all clinical actions, with a few exceptions: actions involving time stamp data (e.g. uterine massage within 15 minutes of delivery) were missing for between 17% and 21% of cases, data on the use of a partograph during labor were missing in 22% of cases (typically because observers failed to observe whether the partograph had actually been filled during labor or had been filled retrospectively), data on preparation of uterotonic for use after delivery were missing in 6% of cases, and data on whether providers asked patients about past complications during the initial exam were missing in 37% of cases (due to a change in the form during the data collection process). For PPH management indicators, data were missing in 0-2% of cases across all indicators, with the exception of uterine packing, blood grouping and cross-matching, and tranexamic acid (missing in 44%, 29%, and 11% of cases, respectively).

We conducted two sensitivity analyses to assess the robustness of our results to different assumptions about missing data. First, we assumed that, if a section was observed, then all actions with missing information within that section were not completed. This is likely to under-estimate guideline adherence. Second, we used multiple imputation to impute missing information conditional on covariates [1,2]. This is likely to improve on the main analysis if, instead of being missing completely at random, data are missing conditional on the covariates included in the imputation model. For example, data may be more likely to be missing from a particular time of day, and time of day may be relevant for the value that the data would have taken had they not been missing. We conducted multiple imputation within each set of clinical actions (risk assessment, prevention, monitoring, and PPH management), and included the following covariates: facility fixed effects, other clinical actions in the section, day of the week, and time of the day. For the PPH section, we included the diagnosed cause of PPH (and omitted the day of the week and the time of the day, because the model would not converge if all of these parameters were included). We imputed five datasets and combined results across the imputations using Rubin’s rules [3]. Third, for variables that rely on time stamps (e.g. the portion of cases in which prophylactic uterotonics were given within one minute of delivery), we calculate an upper bound based on the assumption that cases with missing timing information were done within the recommended time. Finally, for postpartum monitoring, we report the total times patients were monitored (by length-of-stay) as a way to include incidents of monitoring that did not have time stamps. Results from missing data sensitivity analyses are presented in Table S2. Results on postpartum monitoring by women’s length-of-stay are presented in Table S3.

**References**

1. Honaker J, King G, Blackwell, Matthew. Amelia II: A program for missing data. J Stat Softw. 2011;45.7:1–47.

2. King G, Honaker J, Joseph A, Scheve K. Analyzing Incomplete Political Science Data: An Alternative Algorithm for Multiple Imputation. Am Polit Sci Rev. 2001 Mar;95(1).

3. Rubin D. Multiple imputation for nonresponse in surveys. 1987th ed. New York: Wiley;
